# Remote ischaemic conditioning reduces infarct size in animal *in vivo* models of ischaemia-reperfusion injury: a systematic review and meta-analysis

**DOI:** 10.1093/cvr/cvw219

**Published:** 2016-12-17

**Authors:** Daniel I. Bromage, Jack M. J. Pickard, Xavier Rossello, Oliver J. Ziff, Niall Burke, Derek M. Yellon, Sean M. Davidson

**Affiliations:** The Hatter Cardiovascular Institute, University College London, 67 Chenies Mews, London, WC1E 6HX, UK

**Keywords:** Preconditioning, Infarct size, Ischaemia, Reperfusion, Meta-analysis

## Abstract

**Aims:**

The potential of remote ischaemic conditioning (RIC) to ameliorate myocardial ischaemia-reperfusion injury (IRI) remains controversial. We aimed to analyse the pre-clinical evidence base to ascertain the overall effect and variability of RIC in animal *in vivo* models of myocardial IRI. Furthermore, we aimed to investigate the impact of different study protocols on the protective utility of RIC in animal models and identify gaps in our understanding of this promising therapeutic strategy.

**Methods and results:**

Our primary outcome measure was the difference in mean infarct size between RIC and control groups in *in vivo* models of myocardial IRI. A systematic review returned 31 reports, from which we made 22 controlled comparisons of remote ischaemic preconditioning (RIPreC) and 21 of remote ischaemic perconditioning and postconditioning (RIPerC/RIPostC) in a pooled random-effects meta-analysis. In total, our analysis includes data from 280 control animals and 373 animals subject to RIC. Overall, RIPreC reduced infarct size as a percentage of area at risk by 22.8% (95% CI 18.8–26.9%), when compared with untreated controls (*P* < 0.001). Similarly, RIPerC/RIPostC reduced infarct size by 22.2% (95% CI 17.1–25.3%; *P* < 0.001). Interestingly, we observed significant heterogeneity in effect size (T2 = 92.9% and I2 = 99.4%; *P* < 0.001) that could not be explained by any of the experimental variables analysed by meta-regression. However, few reports have systematically characterized RIC protocols, and few of the included *in vivo* studies satisfactorily met study quality requirements, particularly with respect to blinding and randomization.

**Conclusions:**

RIC significantly reduces infarct size in *in vivo* models of myocardial IRI. Heterogeneity between studies could not be explained by the experimental variables tested, but studies are limited in number and lack consistency in quality and study design. There is therefore a clear need for more well-performed *in vivo* studies with particular emphasis on detailed characterization of RIC protocols and investigating the potential impact of gender. Finally, more studies investigating the potential benefit of RIC in larger species are required before translation to humans.

## 1. Introduction

Myocardial ischaemia-reperfusion injury (IRI) describes the deleterious consequences of several pathological processes and cardiac interventions. Most commonly, it is caused by thrombotic occlusion of the coronary artery in ST-segment elevation myocardial infarction (STEMI) and subsequent reperfusion by primary percutaneous coronary intervention (PPCI), but it may result from a range of elective and emergent causes of myocardial ischaemia, including cardiopulmonary bypass and spontaneous reperfusion of STEMI. Despite constantly improving medical and surgical practice, myocardial IRI remains associated with significant morbidity and mortality. For example, in STEMI and despite PPCI, 30 day, 1 year, and 5 year cardiac mortality remains 7.3%, 8.4%, and 13.8%, respectively.^1^

It has been demonstrated that myocardium can be protected from lethal IRI by the application of multiple brief cycles of ischaemia and reperfusion to an organ or tissue remote from the heart, either before, during, or after the index ischaemia (preconditioning, perconditioning, or postconditioning, respectively). Limb remote ischaemic conditioning (RIC) is a cheap, non-invasive intervention that, since its inception in 1997,^2^ has been successfully demonstrated in several pre-clinical studies of myocardial IRI. Subsequently, several Phase II, proof-of-concept clinical studies have translated these findings to variety of clinical settings, albeit frequently using cardiac enzymes as surrogate markers of cellular injury, including coronary artery bypass surgery (CABG),^3–6^ elective abdominal aortic aneurysm repair,^7^^,^^8^ elective cervical decompression surgery,^9^ elective PCI,^10^ and in PPCI for STEMI.^11–13^

However, more recent, large clinical-endpoint studies of RIC in cardiac surgery have been neutral.^14^^,^^15^ Although cardiac surgery may be an inappropriate setting for RIC, given the small peri-operative injury and lack of injurious warm ischaemia-reperfusion,^16^ these findings have prompted an interrogation of the pre-clinical evidence base for RIC and a perceived lack of systematic pre-clinical characterization of the optimal RIC stimulus.^16^ This is in contrast to direct ischaemic conditioning that, despite being limited by the necessity to intervene before the index ischaemia, has been thoroughly characterized.^17^

Well-designed animal studies can provide useful information on relevant factors that may influence outcome. This comprehensive systematic review and meta-analysis scrutinizes basic studies of RIC in *in vivo* animal models of myocardial IRI. Our aim was to ascertain the overall effect and variability of RIC in this context, compared with control (sham procedure or no treatment). We further aimed to investigate determinants of efficacy, including variables such as RIC protocol and use of supplementary oxygen. Our hypothesis was that study quality and publication bias would result in over-estimation of the effect size associated with RIC.^18^^,^^19^

## 2. Methods

### 2.1 Systematic review

The systematic review was performed in accordance with Preferred Reporting Items for Systematic reviews and Meta-Analyses (PRISMA) guidelines.^20^ A literature search was conducted on 21 August 2015 by J.P. Keywords and MeSH terms were used to search Medline and Embase (via OVID) between 1997 and present, and further studies were identified by consultation with experts in the field. Details of the search strategy are available in the Supplementary material online, *Search strategy* section.

Study eligibility criteria were defined using the PICOS approach.^21^*In vivo* animal studies were included and were eligible if they investigated the effect of limb RIC (pre, per, or post) vs. a control (sham procedure or no treatment) on myocardial infarct size (IS), as measured using tetrazolium chloride (TTC),^22^ in any mammalian species, regardless of study design. Transient infra-renal aortic occlusion was considered as bilateral hind limb ischaemia.

Studies were excluded if they did not include or report absolute myocardial IS as a percentage of area at risk (AAR, defined as the myocardial tissue within the vascular territory that is distal to occluded artery and, if not reperfused, is at risk of irreversible ischaemic death).^23^ The AAR varies depending on the exact position of the LAD suture and variable LAD anatomy. IS has a strong positive correlation with AAR and therefore, without correction, a small AAR could create false-positive results for cardioprotection, and vice versa.^24^ Furthermore, studies were excluded if they specifically investigated only the ‘second window’ of cardioprotection (RIC to infarction interval > 1 h),^25–27^ if RIPostC was initiated more than 10 min after reperfusion (after which it is generally believed unlikely to be effective),^28^ if the animals had co-morbidities, if IS was only measured using a method other than TTC, or if they investigated the impact of RIC in the context of heart transplant. Groups in which RIC was administered in combination with another conditioning protocol (local conditioning, for example), or with pharmacological treatments known to have cardioprotective effects, were excluded. Finally, studies investigating neonatal animals were excluded to ensure clinical relevance to IRI.

Reports were excluded if they were not available in English and a publication date restriction of 1997–present was imposed in view of the first publication of the efficacy of RIC in the limb.^2^ Review articles, abstract articles, unpublished material, and ongoing studies were excluded.

Retrieved records were screened for eligibility using the title and abstract, followed by the full text. Eligibility assessment was performed independently in an un-blinded, standardized manner by D.B. and J.P. Disagreements were resolved by examining the full text of the article or by consensus between reviewers in all cases.

Data were independently extracted by two authors (D.B. and J.P.) using pre-defined data fields. Disagreements were resolved by consensus in all cases. We attempted to acquire key missing information by contacting the report authors by e-mail.

Variables for which data were sought were developed using the PICOS approach.^4^^,^^21^ Data items were chosen according to experimental variables with evidence for an effect on myocardial IRI as these were considered likely to impact on the efficacy or RIC, and variables that we considered of potential importance. Full list of data items and assumptions is available in the Supplementary material online, *Data items* section.

### 2.2 Meta-analysis

We defined the primary outcome as the weighted (unstandardized) mean difference (WMD) between ISs in the RIC and control groups. WMD was used as all data were presented in the same units and it gives a biologically relevant value. In each publication, we identified all independent comparisons of IS/AAR% in RIC vs. control groups. Where a study made multiple comparisons to the same control group, the size of the control group was corrected for the number of comparisons made (*n*/number of comparisons).^29^ The secondary outcome was the effect of five pre-defined experimental variables, which we considered most likely to impact on the efficacy of RIC, on WMD.

Comparisons were grouped according to their use of either remote ischaemic preconditioning (RIPreC) or remote ischaemic perconditioning and postconditioning (RIPerC/RIPostC). This is due to temporal differences in their application that, despite not necessarily occurring via different mechanistic pathways,^30^ have different clinical utility. Specifically, RIPerC/RIPostC are clinically applicable to STEMI, whereas RIPreC is not. Subsequent analysis was performed for each group separately.

For each independent comparison, we calculated the effect size as a raw difference in IS/AAR% means (the mean of the control groups minus the mean of the experimental group) and the corresponding 95% confidence interval (CI). To account for anticipated heterogeneity, we pooled effect sizes using random-effects meta-analysis, which considers the within-study and between-study variability and weights each study accordingly. Heterogeneity was quantified using I2 and T2 statistics.^29^^,^^31^ Studies with missing data on any of the pre-defined experimental variables were excluded from the meta-analysis.

Subgroup analyses were performed using univariate meta-regressions to explore which experimental factors and quality indicators contribute to heterogeneity. The percentage of between-study variance explained by variables of interest was assessed using the T2 and adjusted R2 statistics. The significance level was adjusted according to the number of comparisons using the Holm–Bonferroni method and results were considered significant when *P* < 0.01.^32^ Classification of subgroups is given in the Supplementary material online, *Summary measures* section.

Sensitivity analysis was performed to assess the robustness of our findings by performing an additional analysis for both the primary and the secondary endpoints using the standardized mean difference (SMD; the mean of the control group minus the mean of the RIC group, divided by the pooled SD of the two groups). We performed a stratified meta-analysis by subgroup to validate the results obtained by meta-regression.

All analyses were pre-specified and performed using STATA/SE, version 13.1 (StataCorp, College Station, TX, USA); GraphPad Prism version 5.00 for Windows (La Jolla, CA, USA) was used in the production of figures.

### 2.3 Risk of bias

We used a component approach to assess study quality, based on the study report, using the ‘Animal Research: Reporting of *In Vivo* Experiments’ (ARRIVE) guidelines and a 12-item quality score.^33–35^ The derivation of the 12-item quality score is given in the Supplementary material online, *Table S3*; and the methodological features evaluated for each study are given in the Supplementary material online, *Tables S4* and *S5*. Study quality was assessed independently from data extraction and between assessors in an un-blinded, standardized manner by two reviewers (O.Z. and N.B.). Disagreements were resolved by consensus in all cases. We assessed the relationship between study quality and the overall effect of RIC on IS/AAR% using meta-regression, as described above.

Potential publication bias was assessed by visual inspection of a funnel plot for asymmetry, and Egger’s regression analysis for small study effects. No protocols were available with which to examine for selective reporting; however, the methods and results sections of all included studies were carefully compared for inconsistencies.

## 3. RESULTS

### 3.1 Study selection

**Figure 1:**
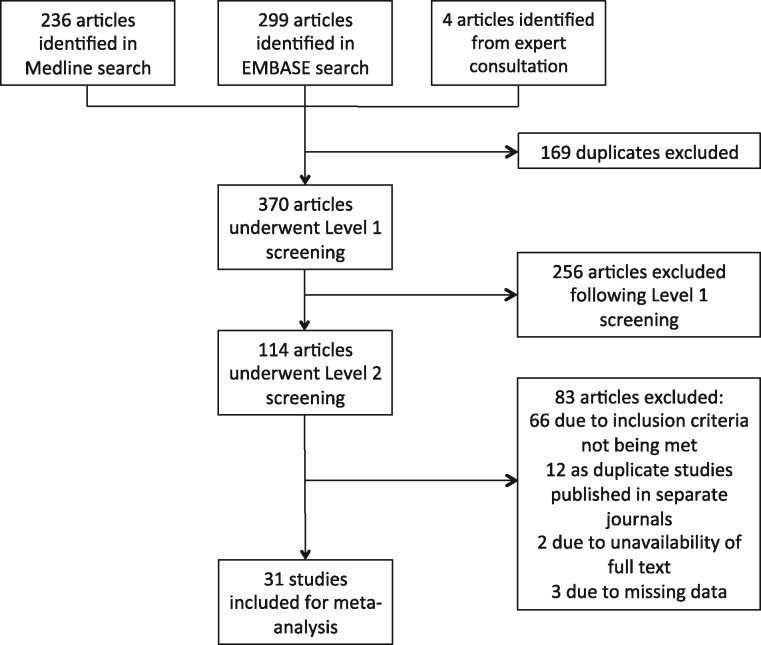
Flow chart of the study selection process. A systematic review yielded 539 reports. After removal of duplicates and the application of inclusion and exclusion criteria, 31 studies were included in the meta-analysis.

Our search returned 539 records, including 169 duplicate reports (consisting of reports returned by both Medline and Embase). In total, 370 reports underwent title and abstract screening, which resulted in 256 exclusions. The remaining 114 reports were retrieved for detailed full text evaluation. Eighty-three articles were excluded, 66 due to failing to meet the inclusion criteria, 12 were abstracts, and two were not retrievable. Of the remaining 34 reports (studies), three were missing data on one or more important experimental variables that we were unable to retrieve by contacting the study authors, and were consequently excluded. These studies are referenced in the Supplementary material online, *References* section. The remaining 31 studies were included in the quantitative synthesis (*Figure 1*). All included studies and their main characteristics are reported in the Supplementary material online, *Data extraction table*.

### 3.2 Meta-analysis

**Figure 2:**
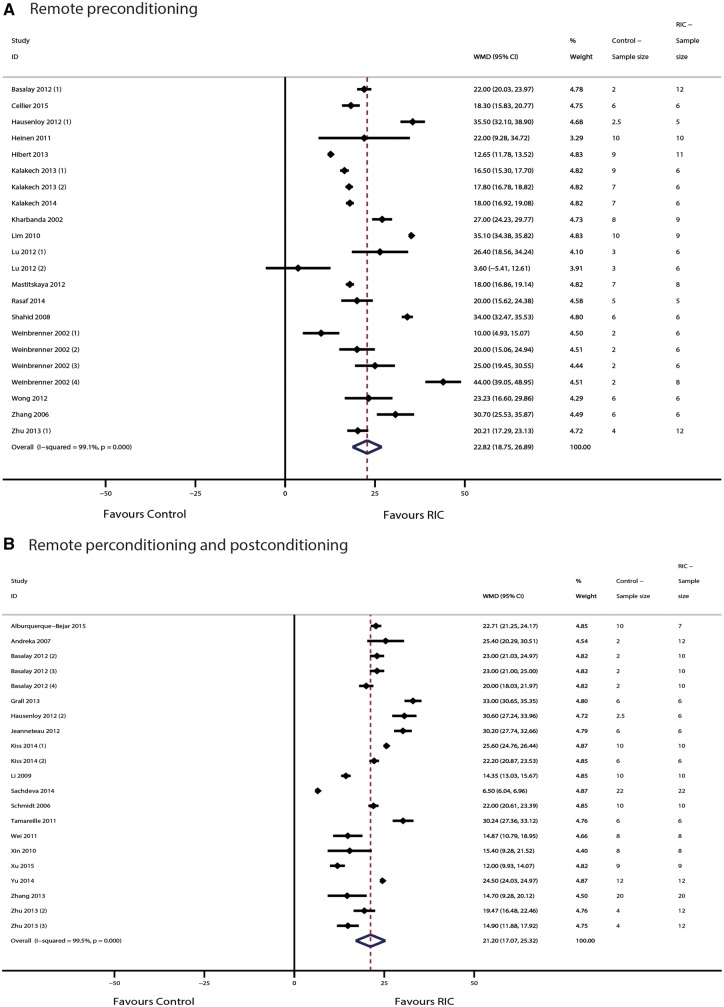
Forest plots of meta-analysis of conditioning efficacy in (*A*) RIPreC and (*B*) RIPerC/RIPostC. Forest plots of the effect of (*A*) RIPreC and (*B*) RIPerC/RIPostC on IS/AAR%, pooled using random-effects meta-analysis; 22 and 21, respectively, controlled comparisons were included, amounting to data from 280 control animals and 373 animals undergoing RIC.

From the 31 included reports, we extracted data on 43 controlled comparisons of RIC in models of myocardial IRI. These were split into 22 comparisons investigating RIPreC, and 21 comparisons investigating RIPerC/RIPostC. In total, our analysis includes data from 280 control animals and 373 animals undergoing RIC. In the RIPreC group, conditioning reduced IS/AAR% by 22.8% (95% CI 18.8–26.9%) when compared with untreated controls (*P* < 0.001; *n* = 22 comparisons, *Figure 2A*). Significant heterogeneity was observed (T2 = 89.2 and I2 = 99.1%; *P* < 0.001). In the RIPreC/RIPostC group, conditioning reduced IS/AAR% by 22.2% (95% CI 17.1–25.3%) when compared with untreated controls (*P* < 0.001; *n* = 21 comparisons, *Figure 2B*). Again, significant heterogeneity was observed (T2 = 90.9 and I2 = 99.5%; *P* < 0.001).

**Figure 3:**
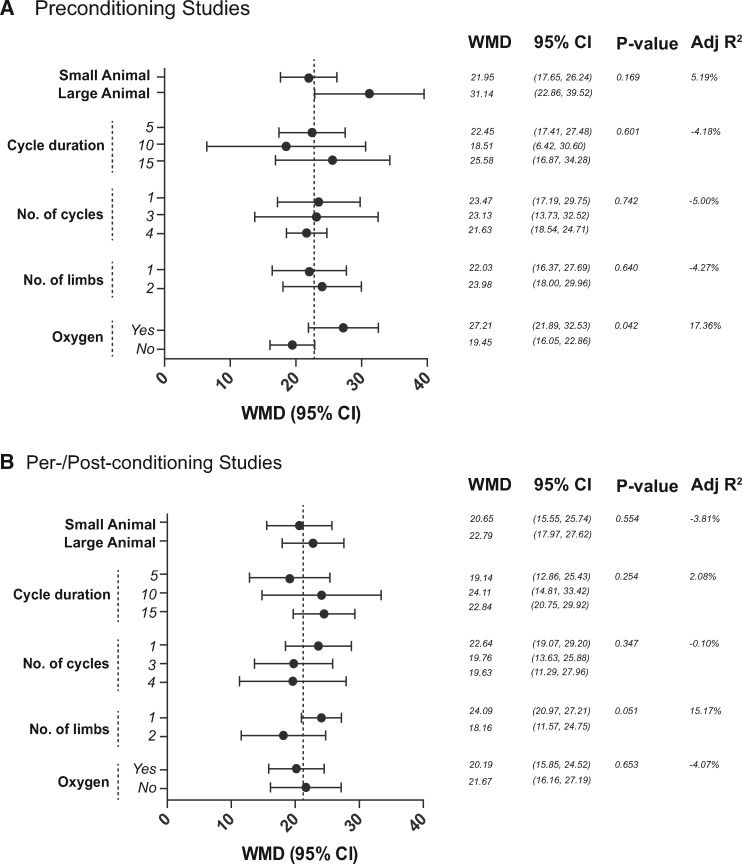
Impact of experimental factors on the efficacy of (*A*) RIPreC and (*B*) RIPerC/RIPostC. WMD and the corresponding 95% CI for each variable were obtained by subgroup stratification. However, the reported *P*-value was obtained by meta-regression to reduce false-positive findings. Studies that used either mice or rats were grouped as ‘small animals’, and those using rabbits or pigs were grouped as ‘large animals’. A *P***-**value of < 0.01 was considered significant.

We investigated potential experimental sources of the observed heterogeneity using meta-regression analysis with IS/AAR% as the dependent variable, and did not find any significant associations with efficacy of RIC (*Figure 3A and B*).

### 3.3 Risk of bias

**Figure 4:**
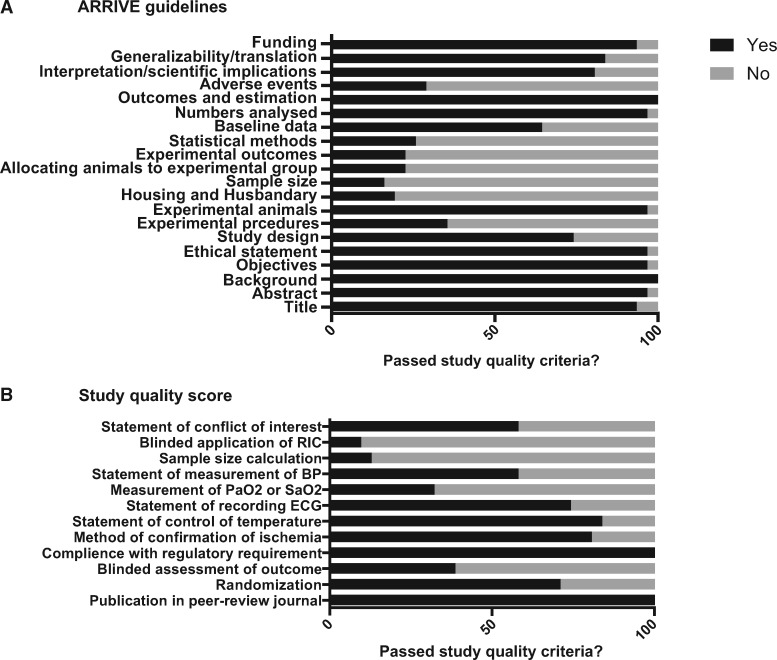
Reporting of study quality indicators. Study quality was assessed using the ARRIVE guidelines on reporting *in vivo* experiments (*A*) and a 12-item quality score (*B*). Values are expressed as the percentage of studies reporting each quality indicator.

Reports achieved a median ARRIVE guidelines score of 14 (inter-quartile range 12–14) out of 20 and a median 12-item quality score of 7 (inter-quartile range 6–8; *Figure 4*). A full breakdown of the scores is given in the Supplementary material online, *Tables S7* and *S8*. Meta-regression indicated that study quality according to either the ARRIVE guidelines score or to a 12-item quality score was not associated with the overall effect (*P* = 0.317 and *P* = 0.846, respectively).

Overall, studies performed particularly poorly in several important areas of experimental design. For example, with respect to the ARRIVE guidelines only 16% reported sample size calculation, 23% reported randomization of animals to experimental groups, 23% defined the primary experimental outcome, and 29% reported detail of adverse events during experiments (*Figure 4A*). Regarding the 12-item quality score, only 32% of studies reported measurement of blood oxygen saturation, 13% reported measurement of blood pressure during the *in vivo* protocol, and 10% performed blinded application of the conditioning protocol (*Figure 4B*). Notably, only 38% of studies performed a blinded assessment of outcome.

**Figure 5:**
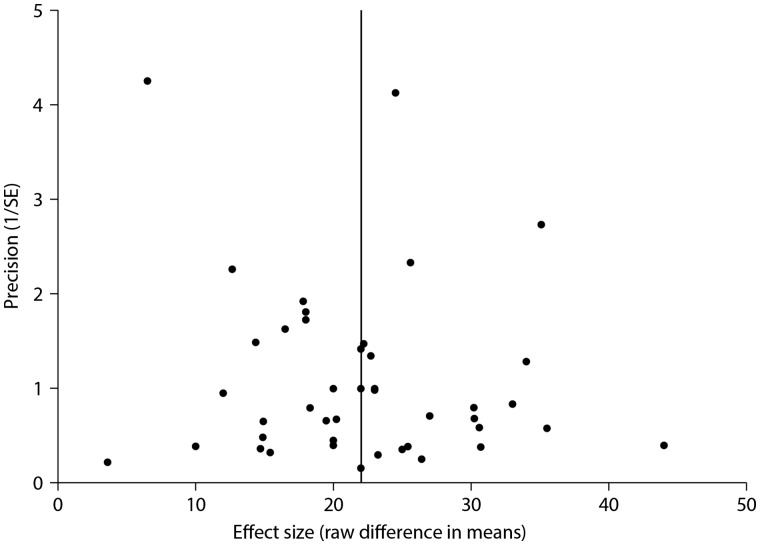
Assessment of publication bias. A funnel plot comparing treatment effect to a measure of study size (precision of the effect estimate). The vertical line represents the mean effect size. This plot was assessed visually, with further analysis of publication bias performed using Egger’s regression test.

The impact of publication bias on the overall effect was assessed by visual analysis of the funnel plot, which suggested that small and negative studies might be under-represented (*Figure 5*). However, in Egger’s regression test the null-hypothesis of no small-study effect was not rejected at *P* = 0.216 (estimated bias coefficient 3.75 ± 2.98 SE).

### 3.4 Sensitivity analysis

When re-running our analysis using the SMD, all results were similar to those found using the WMD. We found a highly significant (*P* < 0.001) overall effect of RIPreC (SMD of 11.06; 95% CI 8.52–13.60), as well as a similar level of heterogeneity (*I*^2 ^=^ ^91.1%). None of the experimental variables was significant after correction by multiple comparison. For RIPerC/RIPostC, all results using the SMD were likewise similar to those found using the WMD. We found a highly significant (*P* < 0.001) overall effect (SMD of 13.14; 95% CI 10.51–15.77) and similar heterogeneity (*I*^2 ^=^ ^93.3%). None of the experimental variables was significant after correction by multiple comparison.

## 4. Discussion

Our major finding is that both RIPreC and RIPerC/RIPostC have a large beneficial effect of both on IS. This finding is based on a comprehensive systematic review, including over 650 animals. However, there were relatively few pre-clinical studies investigating RIC and even fewer systematically characterizing the protocol, which limited our analysis.

Furthermore, we found inconsistency in the design of pre-clinical RIC studies with very few that were randomized effectively, included both male and female animals, and were double blinded with respect to procedure and outcome, which highlights the need for further well-designed, pre-clinical studies of RIC.

These are important findings in the context of recent pessimism regarding RIC as a genuine cardioprotective phenomenon.^36^^,^^37^ Clinical trials investigating the efficacy of RIC in myocardial IRI have had mixed results,^11^^,^^14^ which has been attributed to clinical variables, including propofol administration.^36^ However, each of the factors described above may influence outcome through lack of generalizability or unconscious bias,^19^ and therefore indirectly impact upon attempts to translate RIC to humans in clinical trials.^16^

### 4.1 Determinants of RIC

Interestingly, we found high levels of heterogeneity between studies. To investigate whether study protocol could account for the observed heterogeneity, and to elucidate the determinants of efficacy of RIC, we assessed the impact of experimental variables on effect size using a meta-regression analysis. This approach has been successfully applied to several promising pre-clinical interventions to date. For example, Lim *et al.*^38^ were able to demonstrate that ciclosporin was not effective at limiting myocardial IS in pig models of IRI, compared with small animal models. This finding might be important in the context of a subsequent neutral clinical study of ciclosporin before reperfusion in patients with STEMI.^39^ Therefore, finding the parameters responsible for heterogeneity can guide pre-clinical and clinical study design.

However, none of the tested experimental variables, which included species, cycle duration, number of cycles, number of conditioned limbs and the use of supplementary oxygen, was associated with effect size. In a recent analysis of pre-clinical studies of local ischaemic preconditioning, which included limited analysis of RIC, species (rodent vs. non-rodents) accounted for a substantial amount of the observed heterogeneity.^17^ Although we found no significant association between animal size (rodents vs. non-rodents), there was a pattern of increased efficacy of RIPreC in large animals.

Wever *et al.*^17^ also describe no association between effect size and the number, timing and duration of cycles, which is reflected in this study. Specifically, we found 1, 3 and 4 cycles to be equally effective. Conversely, in one of the few neutral comparisons in our analysis, Lu *et al.*^40^ utilized a protocol consisting of one cycle of 5 min RIPreC. Interestingly, this amounts to the lowest total ischaemic ‘dose’ (a function of cycle number and duration) of any study of RIPreC. Amongst the RIPerC/RIPostC studies, only Li *et al.*^41^ applied one cycle of 5 min limb ischaemia and reported a relatively modest, albeit significant, effect (WMD 14.35%, 95% CI 13.03–15.67%). Others, including Mastitskaya *et al.*,^42^ have applied a single cycle of longer duration and achieved greater protection. It is therefore plausible that ischaemic ‘dose’ or burden, rather than cycle number or duration alone, is the dependent variable, but very few studies have examined this systematically *in vivo*. An exploratory analysis of the present data demonstrated no such association (data not shown); however this was limited by a narrow distribution of total ischaemic times in our dataset. Interestingly, a dose-dependent effect of RIPreC has been observed, with greater cardioprotection after 10 or 15 min compared with 5 min continuous infra-renal aortic occlusion.^43^ In a study comparing bilateral and unilateral RIPreC in protection against renal IRI, bilateral was found to be more effective.^44^ Taken together, this may suggest that total ischaemic dose is important, but this needs testing in specifically designed experiments.

Furthermore, there may be an upper limit to what is an effective ischaemic dose. A recent characterization of the RIC protocol *ex vivo* reported that four and six, but not eight, cycles of RIC were protective in mice.^45^ Similarly, although 5 min or 10 min limb ischaemia protected against subsequent liver injury, 30 min and 60 min actually increased injury.^46^ This issue is worthy of further attention, particularly using a more realistic setting of aged animals with co-morbidities. This may suggest a therapeutic window for RIC but there is very limited pre-clinical evidence, in contrast to direct ischaemic conditioning where the number of cycles is demonstrably important,^17^ and it is clear that detailed characterization of study protocol *in vivo* is urgently necessary in order to answer these questions.

After a period of limb ischaemia, it has been assumed that reperfusion is necessary, either to wash out the putative humoral factor or because reperfusion-induced ROS may be necessary to activate signalling pathways. Additionally, some studies have suggested that reactive hyperaemia is an important factor in the response.^47^ However it is worth bearing in mind that the original description in rabbits, where RIPreC was achieved by 55–65% stenosis of the femoral artery in combination with rapid electrical stimulation of the gastrocnemius muscle for 30 min, did not actually involve reperfusion.^2^ This appears to support the hypothesis that multiple mechanisms are involved.^48^

Similarly, our analysis supports the finding of Wever *et al.*^17^ of no association with the number of limbs conditioned. Johnsen *et al.*^45^ further reported that 2 and 5 min, in contrast to 10 min, cycles were beneficial in mice. This remains consistent with the concept of a therapeutic window of RIC but there is a paucity of *in vivo* experimental data relating to the precise RIC protocol. Our analysis demonstrated 10 min cycles to be equally effective as 5 min cycles, albeit only in larger species as no studies in our meta-regression used 10 min cycles in mice. We also noticed a pattern of reduced efficacy in RIPreC/RIPostC studies using two limbs instead of one, which may exceed the therapeutic window. However, this finding was not statistically significant and should only be considered hypothesis-generating.

The use of supplementary oxygen in acute myocardial infarction is controversial,^49^ but the role of supplementary oxygen in RIC has not, to our knowledge, been investigated. It could be hypothesized that RIC is driven by cellular hypoxia in the conditioned limb which, at least in part, might be alleviated by ventilation with supplementary oxygen. However, despite finding the conditioning of animals with and without oxygen to be equally effective, there was a pattern of increased efficacy in animals ventilated with supplementary oxygen in the RIPreC comparisons. This has not been specifically investigated but our results would suggest further investigation is warranted.

Furthermore, there is evidence that the degree of cardioprotection conferred by RIC is proportional to the duration of index ischaemia. Specifically, Kleinbongard *et al.*^50^ demonstrated longer ischaemic times to be associated with a greater efficacy of RIC, albeit in a study of patients undergoing CABG, which may be related to a greater target for protection. In an exploratory analysis, we found no statistical effect of index ischaemic time (see Supplementary material online, *Table S5* and *S6*); however, there was a pattern of reduced efficacy of RIC at longer ischaemic durations in RIPreC but not RIPerC/RIPostC. This might suggest an important role for the timing of intervention, but should be interpreted with caution in view of the limited number of comparisons available after stratification according to study protocol.

There were several experimental variables that were of considerable interest but that lacked sufficient power for statistical analysis. For example, it has been suggested that propofol interferes with the development of RIC,^51^^,^^52^ and it has been implicated as a potential reason for the apparent lack of translation of RIC.^53^ Interestingly, we observed studies where propofol (together with either opioid analgesia ± pancuronium) was administered to have effect sizes above the mean.^54^^,^^55^

Furthermore, studies reporting the use of mixed gender experimental groups reported apparently smaller effect sizes, and one of the few neutral studies was performed only in female animals.^56^ Gender can potentially impact upon IRI possibly due to the cardioprotection conferred by oestrogen,^57^^,^^58^ and potential temporal variability in cardioprotection as a result of the oestrous cycle of female rats.^59^ However, investigation of the role of gender is conspicuously absent from pre-clinical studies of RIC and these qualitative data require further testing in formal, well-designed studies.

An important result of this study is the need for more pre-clinical characterization of RIC, which as well as the aforementioned variables should include the potential role of occlusion technique (arterial clamping vs. external compression), which may be important due to the possible involvement of limb collaterals,^60^ the putative effect of shear stress in RIC,^47^ and the role of pre-treatment with heparin, which has been reported to be cardioprotective in the context of IRI.^61–65^ Likewise, it has been hypothesized that the interval between the conditioning stimulus and the onset of index ischaemia might be an important determinant of the efficacy of remote preconditioning.^16^

A further consideration is the impact of co-morbidities and co-medications on the efficacy of RIC. The animal models used in pre-clinical studies frequently do not reflect the complex risk factor, co-morbidity and pharmaceutical profile of humans with cardiovascular disease,^66^^,^^67^ which may impede the development of RIC. For example, Jensen *et al.*^68^ treated isolated perfused rabbit hearts subjected to IRI with plasma dialysate from diabetic patients treated with RIC. They were unable to confer cardioprotection using dialysate from patients with peripheral neuropathy compared with non-diabetic patients and diabetic patients without neuropathy. Baranyai *et al.*^69^ similarly found that acute hyperglycaemia abrogated the beneficial effect of RIPerC in Wistar rats. However, despite considerable investigation of these features in local ischaemic preconditioning (reviewed by Ferdinandy *et al.*^70^), there is little experimental data relating to these factors in the context of RIC. Indeed, animals with co-morbidities were specifically excluded from the present analysis due to the small number of studies available. Nonetheless, co-morbidities should be considered when investigating the optimum protocol for the delivery of RIC.

### 4.2 Risk of bias

Poor methodological quality and publication bias can result in over-estimation of effect size.^71–73^ In turn, this can engender enthusiasm about the benefit of a treatment where, in fact, none exists. It is therefore essential to examine the impact of study quality on size of effect, to which end we found no statistical relationship using meta-regression.

However, we made some interesting observations. Aspects of the report relating to the experimental procedure, including control of temperature and recording of the ECG, were generally well reported. However, there was generally poor observation of the ARRIVE guidelines, particularly in relation to reporting of sample size calculations, randomization, blinding and adverse events, which can result in selective exclusion. For example, in an interesting meta-analysis of systematic reviews, Hirst *et al.*^74^ reported that failure to randomize significantly increased effect size. Furthermore, appropriate monitoring of experimental animals, including recording of blood oxygen saturation and blood pressure, was poorly reported. These facets are clearly essential to ensure good quality research and were a central tenet of a recent position paper on improving the pre-clinical assessment of novel cardioprotective therapies.^19^ In some cases, these omissions will represent inadequate reporting but in others it is likely that these crucial elements of study design were not performed. We elected not to analyse each quality criterion independently due to insufficient reporting and to avoid false**-**positive findings due to multiple comparisons. However, poor adherence to certain quality criteria may account for the heterogeneity observed in this meta-analysis.

Finally, our assessment of publication bias by visual analysis of the funnel plot suggested that small, neutral studies may be under-represented; however, this did not statistically impact on the overall effect size, which is reassuring.

### 4.3 Limitations

The validity of this meta-analysis is contingent upon the quality of reporting of the included studies. Unpublished studies and those with missing data could not be included in the meta-analysis, and others did not meet important quality criteria including poor information regarding statistical analysis and blinding. However, the absence of a statistical impact from study quality or publication bias is reassuring in this regard. We were limited by being unable to consider manuscripts not available in English and we acknowledge that we did not carry out a systematic literature review to determine which experimental variables to include in our analyses, which might be subject to selection bias. A relatively small number of studies were included in the meta-analysis, thereby limiting the power of the study, which was further affected by multiple comparisons within individual studies; however, we elected to include all comparisons to avoid selection bias. Meta-regression is inherently limited; however, to ensure this was as robust as possible we performed a stratified meta-analysis by subgroup that yielded similar results, including a highly significant overall effect of RIC, significant heterogeneity, and no effect of any of the experimental variables we included in our model.

## 5. Recommendations and conclusions

This systematic review and meta-analysis of pre-clinical *in vivo* studies of myocardial IRI demonstrates a significant and highly reproducible beneficial effect of RIC. This effect was highly heterogeneous, a finding that may be due to un-measurable, multifactorial differences between individual experimenters and laboratories. However, importantly, *in vivo* studies to date suggest the optimal RIC stimulus has not yet been identified. There was a paucity of pre-clinical characterization of the RIC protocol and poor reporting of quality indicators. This is important not so the protocol can be translated to humans, but in order to understand the important parameters and/or markers that will facilitate optimization of the protocol in humans.

There has been a great deal of debate regarding neutral clinical studies of RIC.^16^^,^^36^ However, before we try to understand these failings and design future studies it is essential to fully describe RIC in pre-clinical experiments. At present, studies variably (and apparently randomly) apply the intervention to one or two limbs, for varying periods of time, with a variable number of cycles and with inconsistent timing with respect to the injurious ischaemic episode. To this end, we have identified a need for more, well-performed studies with a focus on characterization rather than detailed elucidation of mechanisms. In particular, these should concentrate on investigating the potential impact of gender and the number, timing and duration of cycles on the efficacy of RIC. These aims would be greatly aided by the identification of a biomarker or a critical physiological parameter(s) that correlates with protection. More studies investigating the potential benefit of RIC in larger species are required before translation to humans and we highlight the potential role of supplementary oxygen as particularly interesting for exploration. Finally, future research should focus on investigating other potential reasons for neutral clinical studies of RIC, including co-morbidities and adjunctive therapies.

## Supplementary material

Supplementary material is available at *Cardiovascular Research* online.

## Supplementary Material

Supplementary Data
