# Supplementary material for: Remote ischaemic conditioning reduces infarct size in animal in vivo models of ischaemia-reperfusion injury: a systematic review and meta-analysis
Source: Cardiovasc Res. 2016 Dec 17;113(3):288–97. doi: 10.1093/cvr/cvw219 (PMC5408955; doi:10.1093/cvr/cvw219)
Supplement: Supplementary Data [file cvw219_Supplementary_Data.zip › Systematic_review_supplementary_results_new.docx]

SUPPLEMENTARY MATERIAL

Our primary outcome, data sources, eligibility criteria, data extraction and data synthesis methods were pre-defined and documented in our protocol.

Search strategy

The search strategy was defined in an iterative manner, using previously published guidelines,[^1-3^](#_ENREF_1) by DB and JP and peer reviewed by members of the Hatter Cardiovascular Institute research group. Our search will be limited to reports available in English due to limited time and financial resources for translation. We will use the following search strategy, incorporating keywords and MeSH terms, for MEDLINE:

1. (remote adj2 $condition$).mp.
2. (remote adj isch$emic).mp.
3. remote precondition$.mp.
4. remote postcondition$.mp.
5. remote percondition$.mp.
6. limb $condition$.mp.
7. ripc.mp.
8. limb isch$emi$.mp.
9. $condition$.mp.
10. 8 and 9
11. 1 or 2 or 3 or 4 or 5 or 6 or 7 or 10
12. exp Reperfusion Injury/
13. exp Myocardial Ischemia/
14. Myocardial Infarction/
15. infarct size.mp.
16. ("infarct size" or "size of infarct*").mp.
17. cardioprotection.mp.
18. 12 or 13 or 14 or 15 or 16 or 17
19. 11 and 18
20. Limit 19 to animal
21. Limit 20 to (english language and yr=”1997-Current”)
22. Limit 21 to review articles
23. 21 not 22

Differences in the search strategy between Medline and Embase are described in table S1

| **Search term** | **Database** | |
| --- | --- | --- |
|  | **Medline** | **Embase** |
| Cardioprotection | cardioprotection.mp. (keyword) | heart protection/ (MeSH term) |
| Search review articles | limit 21 to review articles | limit 21 to “reviews (maximizes sensitivity)” |

Table S1: Differences in search strategy between databases

Transplanted hearts were added to the exclusion criteria *post hoc* as it was apparent that these experiments fulfilled our inclusion criteria but were likely to confer additional injury during the transplantation process.

Study selection

Duplicates will be excluded using Endnote for Windows (Thomas Reuters, PA, US). Eligibility assessment will be performed independently in an un-blinded, standardized manner by 2 reviewers (DB and JP). To ensure reliability and improve subjectivity of screening of relevant titles and abstracts, DB and JP will independently screen 10% of a random sample of the search results. The exercise will be repeated until 90% agreement was achieved, before screening all search results. Disagreements between reviewers will be resolved by examining the full text of the article or by consensus. When this fails to resolve the disagreement, attempts will be made to contact authors of the original study by e-mail, where appropriate. Further unresolved conflict will be resolved by arbitration from the senior author. Inter-rater agreement and the frequency of arbitration will be recorded. The full text of eligible records will be retrieved and subjected to full text screening. The same calibration exercise as described will be performed prior to un-blinded, standardized eligibility assessment by DB and JP. Conflicts will be resolved as per the title and abstract screening.

During the title and abstract screening calibration exercise, inter-rater agreement was 94%, with no instances of arbitration or reason to contact the authors of the study. During full text screening, inter-rater agreement was 87%, with no instances of arbitration or reason to contact the authors of the study. The 13% of references upon which the author disagreed were accounted for by screening errors rather than fundamental disagreement about the features of the study.

Data collection process

We have developed a data extraction sheet based on the Cochrane Consumers and Communications Review Group’s data extraction template,[^4^](#_ENREF_4) which will be pilot-tested on ten randomly-selected included studies, and refined accordingly.

Data will be independently extracted by 2 authors (DB and JP) using predefined data fields, including study quality indicators. To ensure reliability and improve subjectivity of data extraction, DB and JP will independently collect data from 10% of a random sample of included studies. The exercise will be repeated until 90% agreement is achieved, before extracting data from all included studies. Disagreements will be resolved by consensus. When this fails, attempts will be made to contact authors of the original study by e-mail, where appropriate. Further unresolved conflict will be resolved by arbitration from the senior author. Inter-rater agreement and the frequency of arbitration will be recorded.

Following data extraction, searches will be conducted based on first and senior author name, sample size and size of outcome to identify double counting. These parameters have been chosen due to expected high completeness and based on recommendations in the PRISMA statement.[^5^](#_ENREF_5) If multiple reports for the same study are identified they will be compared for logical inconsistencies, which will subsequently be accounted for by contacting the report author by e-mail. We will likewise attempt to acquire key missing information in the same way.

Data items

Variables for which data will be sought were developed using the PICOS approach.^4^ [^6^](#_ENREF_6) Data items were chosen according to experimental variables with evidence for an effect on myocardial IRI (see Table S2) as these were considered likely to impact on the efficacy or RIC. Examples of such variables are species and strain, gender, choice of anaesthetic and duration of ischaemia and reperfusion:

Species and strain: An extensive collateral circulation is known to afford cardioprotection and therefore species with extensive collateral circulation, such as dogs, have smaller infarct sizes after IRI relative to rabbits, pigs, rats and mice.[^7^](#_ENREF_7)^,^ [^8^](#_ENREF_8) Additionally, although Sprague-Dawley (SD) is the strain of rat most commonly used for myocardial infarction experiments.[^9^](#_ENREF_9) It has been demonstrated that SD rats have the most marked variability in infarct size and cardiac dysfunction after IRI owing to the least consistent LAD branching pattern of 6 strains tested.[^9^](#_ENREF_9) These factors may impact on the efficacy of RIC.

Gender: Gender can potentially impact IRI due to the cardioprotection conferred by oestrogen,[^10^](#_ENREF_10)^,^ [^11^](#_ENREF_11) and potential temporal variability in cardioprotection as a result of the oestrous cycle of female rats.[^12^](#_ENREF_12)

Choice of anaesthetic: Several anaesthetic agents adversely affect cardiovascular functional indices, which include heart rate, cardiac output, blood pressure and LV dimensions.[^13^](#_ENREF_13) Furthermore, the cardioprotective effects of volatile anaesthetics such as isoflurane are well-described,[^14^](#_ENREF_14) and it has been suggested that propofol interferes with the development of RIC.[^15^](#_ENREF_15)^,^ [^16^](#_ENREF_16)

Duration of ischaemia and reperfusion: Increased ischaemic duration,[^17^](#_ENREF_17) and possibly reperfusion duration,[^18^](#_ENREF_18) have been reported to increase infarct size.

We will also include variables without extensive evidence that we consider of potential importance. Although not all the data items have been investigated in the literature in the setting of cardioprotection, we want to cover a broad range of experimental variables in order to obtain a clear picture of how the efficacy of RIC may be influenced *in vivo*. We have not performed a separate systematic review but we believe we have accounted for all potentially pertinent methodological variables. Any data items added after the review started will be highlighted and justified. All extraction forms will be archived and made available, if required.

| **Category** | **Data item** |
| --- | --- |
| **Study details** | First author, senior author, date, report format (abstract/full), country of origin |
| **Populations/subjects (P)** | Species, strain, age/weight |
| **Interventions (I)** | Pre-/per-/post-conditioning, RIC cycle time, RIC number of cycles, number of limbs, RIC technique (cuff/clamp), time to index ischaemia, index ischaemic duration, coronary territory occluded, reperfusion duration, non-recovery vs. recovery, anaesthetic type, anaesthetic dose (mg/kg), other peri-operative medication, use of anti-coagulant, type of anti-coagulant, intubated/ventilated, supplementary oxygen |
| **Outcomes (O)** | Infarct size as primary endpoint, mean infarct size (%AAR), mean AAR, AAR technique, standard deviation, SEM, other reported statistical analyses (pertinent to infarct) |
| **Study design (S)** | Sample size |

Table S2: Data items, developed using the PICOS approach

We also made the following assumptions:

- The included studies contained several definitions of pre-, per- and post-conditioning. For clarity, despite the terminology used in the report, we defined preconditioning as any stimulus where the last cycle of ischaemia had been completed by the time of index ischaemia, perconditioning as any ischaemic stimulus that overlapped in full or in part with the index ischaemia, and postconditioning as any stimulus that began at or after the time of myocardial reperfusion.
- When a report described ‘femoral artery occlusion’ we assumed this to be with a vascular clamp. Likewise, where hindlimb occlusion was reported we assumed the use of an external cuff or tourniquet. A cuff was defined as an external inflatable device, while a tourniquet described any other kind of external compression. In all cases we assumed the cuff was inflated to an appropriate pressure.
- We assumed any reperfusion duration ≤4 h to be non-recovery and anything over that to be a recovery model.
- We defined a time of >1hr from the last RIC cycle to the onset of index ischaemia as the ‘second window’ of protection. This study was concerned with the acute cardioprotective potential of RIC, thus these studies were excluded from the analysis.
- Descriptions of the LAD and left coronary artery (LCA) were assumed to be interchangeable in rodents.

Summary measures

*Primary outcome measure*

The weighted (unstandardized) mean difference (WMD) between infarct sizes in RIC versus control procedure arms*.* In studies that included additional groups that were, for example, subjected to RIC in addition to another conditioning protocol or to pharmacological treatments known to have cardioprotective effects, only the RIC and control groups (as per our eligibility criteria) were included in the analysis.

*Secondary outcome measures*

The effect of pre-defined experimental variables on WMD. These are defined as:

1. Species: Studies that used either mice or rats were grouped as ‘small animals’, and those using rabbits or pigs were grouped as ‘large animals’.
2. Cycle duration: The reported duration of ischaemia applied to the limb in each cycle. In the analysis, studies were grouped as 5, 10 or 15 minutes of limb ischaemia.
3. Number of cycles: The number of times this ischaemic episode was applied in succession to the limb. Studies were grouped as using 1, 3 or 4 cycles of ischaemia.
4. Number of limbs: The RIC protocol reported by each study was either applied to one or both (i.e. bilateral) limbs. Conditioning by infra-renal aortic occlusion was classed as bilateral hindlimb ischaemia, whereas studies using supra-renal aortic occlusion were excluded.
5. Oxygen: Each study was given a binary score according to its reported use of supplementary oxygen in their ventilation protocol for the *in vivo* procedure. If the study did not mention whether supplementary oxygen was used, it was assumed the animal was ventilated with room air.

Adapted quality score

To assess study quality, we used an adapted quality score in addition to the ARRIVE guidelines to ensure the measure of study quality was appropriate for studies investigating RIC in *in vivo* animal models of IRI. From the original 10-item score published by developed by Macleod *et al.* in response to a perceived failure of translation of promising neuroprotective agents in stroke,[^19-21^](#_ENREF_19) we removed two and added 4 items, resulting in a 12-item custom score which we applied to studies in the meta-analysis. If the monitoring of ST segments or rhythms was reported, we assumed that heart rate monitoring as available

| **Added to Cochrane study quality score** | **Removed from Cochrane study quality score** |
| --- | --- |
| Statement of measurement of PaO2 and SaO2 | Use of animals with co-morbidities |
| Statement of recording of ECG | Blinded induction of ischaemia |
| Statement of measurement of blood pressure |  |
| Blinded application of conditioning protocol |  |

Table S3. Items removed and added from the Cochrane study quality score to make the adapted score used in the study

Data extraction table

| **1** | **2** | **3** | **4** | **5** | **6** | **7** | **8** | **9** | **10** | **11** | **12** | **13** | **14** | **15** | **16** | **17** | **18** | **19** | **20** | **21** | **22** | **23** | **24** | **25** | **26** |
| --- | --- | --- | --- | --- | --- | --- | --- | --- | --- | --- | --- | --- | --- | --- | --- | --- | --- | --- | --- | --- | --- | --- | --- | --- | --- |
| 1 | Pig | M | 25-30kg | Per | 5 | 5 | 4 | 1 | 20 | Clamp | N/A | 40 | LAD | 120 | NR | Y | Propofol, fentanyl | N | SEM | 10 | 59.2 | 3.17 | 7 | 36.5 | 4.7 |
| 2 | Pig | M/F | 18-22kg | Post | 5 | 5 | 4 | 1 | 20 | Cuff | N/A | 90 | LAD | 4320 | R | Y | Ketamine | Heparin | SEM | 12 | 48.4 | 5.2 | 12 | 23 | 2.4 |
| 3 | Rat | M | N/R | Pre | 15 | 10 | 1 | 2 | 30 | Clamp | 10 | 30 | LAD | 120 | NR | N | Pentobarbitone | N | SD | 8 | 46 | 7 | 10 | 19 | 5 |
|  | Rat | M | N/R | Per | 15 | N/A | 1 | 2 | 30 | Clamp | N/A | 30 | LAD | 120 | NR | N | Pentobarbitone | N | SD |  |  |  | 10 | 21 | 7 |
| 4 | Rat | M | Adult | Pre | 15 | N/A | 1 | 2 | 30 | Clamp | 10 | 30 | LAD | 120 | NR | N | Pentobarbitone | N | SEM | 8 | 41 | 2 | 12 | 19 | 1 |
|  | Rat | M | Adult | Per | 15 | N/A | 1 | 2 | 30 | Clamp | N/A | 30 | LAD | 120 | NR | N | Pentobarbitone | N | SEM |  |  |  | 10 | 18 | 1 |
|  | Rat | M | Adult | Per | 15 | N/A | 1 | 2 | 30 | Clamp | N/A | 30 | LAD | 120 | NR | N | Pentobarbitone | N | SEM |  |  |  | 10 | 18 | 2 |
|  | Rat | M | Adult | Post | 15 | N/A | 1 | 2 | 30 | Clamp | N/A | 30 | LAD | 120 | NR | N | Pentobarbitone | N | SEM |  |  |  | 10 | 21 | 1 |
|  | Rat | M | Adult | Post | 15 | N/A | 1 | 2 | 30 | Clamp | N/A | 30 | LAD | 120 | NR | N | Pentobarbitone | N | SEM |  |  |  | 10 | 43 | 3 |
| 5 | Rat | M | Adult | Pre | 5 | 5 | 4 | 1 | 20 | Clamp | 5 | 40 | LAD | 120 | NR | N | Pentobarbitone | N | SEM | 6 | 66.5 | 5.5 | 6 | 48.2 | 5.2 |
| 6 | Rat | M | 8-10 weeks | Per | 10 | 10 | 1 | 1 | 10 | Tourniquet | N/A | 40 | LAD | 120 | NR | N | Pentobarbitone | N | SEM | 6 | 54.9 | 6.01 | 6 | 21.9 | 4.0 |
| 7 | Pig | M | 27-35kg | Pre | 5 | 5 | 4 | 1 | 20 | Clamp | 5 | 60 | LAD | 180 | NR | Y | Propofol, pancuronium, fentanyl | Heparin | SEM | 5 | 48.8 | 4.2 | 5 | 13.3 | 2.2 |
|  | Pig | M | 27-35kg | Per | 5 | 5 | 4 | 1 | 20 | Clamp | N/A | 60 | LAD | 180 | NR | Y | Propofol, pancuronium, fentanyl | Heparin | SEM |  |  |  | 6 | 18.2 | 2 |
| 8 | Rat | M | Adult | Pre | 5 | 5 | 4 | 2 | 40 | Cuff | 10 | 35 | LAD | 120 | NR | Y | Pentobarbitone | N | SD | 10 | 76 | 14 | 10 | 54 | 15 |
| 9 | Rat | M | 8-10 weeks | Pre | 10 | 10 | 1 | 1 | 10 | Clamp | 10 | 40 | LAD | 120 | NR | N | Pentobarbitone | N | SEM | 9 | 64.9 | 2.6 | 11 | 52.2 | 3.7 |
| 10 | Rat | M | 288+/-9kg | Per | 10 | 10 | 1 | 1 | 10 | Tourniquet | N/A | 40 | LAD | 120 | NR | N | Pentobarbitone | N | SEM | 6 | 54.6 | 4.7 | 6 | 24.4 | 5.9 |
| 11 | Rat | M | Adult | Pre | 5 | 5 | 4 | 1 | 20 | Clamp | 5 | 40 | LAD | 120 | NR | N | Pentobarbitone | N | SEM | 7 | 65.3 | 2.9 | 6 | 47.3 | 2.2 |
| 12 | Mouse | M | N/R | Pre | 5 | 5 | 4 | 1 | 20 | Clamp | 5 | 30 | LAD | 120 | NR | N | Pentobarbitone | N | SEM | 9 | 40.6 | 3.6 | 6 | 24.1 | 2.8 |
|  | Rat | M | 2-3 months | Pre | 5 | 5 | 4 | 1 | 20 | Clamp | 5 | 40 | LAD | 120 | NR | N | Pentobarbitone | N | SEM | 7 | 65.1 | 2.7 | 6 | 47.3 | 2.1 |
| 13 | Pig | N/R | 15kg | Pre | 5 | 5 | 4 | 1 | 20 | Tourniquet | 5 | 40 | LAD | 120 | NR | Y | Midazolam, pentobarbitone | Heparin | SEM | 8 | 53 | 8 | 9 | 26 | 9 |
| 14 | Rat | M | N/R | Per | 15 | N/A | 1 | 2 | 30 | Clamp | N/A | 30 | LAD | 120 | NR | N | Pentobarbitone | N | SEM | 10 | 69 | 2 | 10 | 43.4 | 3.8 |
|  | Rat | M | N/R | Per | 15 | N/A | 1 | 2 | 30 | Clamp | N/A | 30 | LAD | 120 | NR | N | Pentobarbitone | N | SEM | 6 | 68.6 | 0.8 | 6 | 46.4 | 4 |
| 15 | Rabbit | M/F | 2.5-3kg | Per | 5 | 1 | 1 | 1 | 5 | Clamp | N/A | 30 | LAD | 180 | NR | Y | Pentobarbitone | Heparin | SD | 10 | 31.5 | 1.3 | 10 | 17.1 | 1.7 |
| 16 | Mouse | M | 10-12 weeks | Pre | 5 | 5 | 3 | 1 | 15 | Clamp | 5 | 30 | LAD | 120 | NR | Y | Ketamine, xylazine, atropine | N | SEM | 10 | 56.7 | 3.2 | 9 | 21.6 | 1.6 |
| 17 | Rat | M | 280-300g | Pre | 5 | 5 | 1 | 1 | 5 | Clamp | 5 | 30 | LAD | 120 | NR | N | Pentobarbitone | N | SD | 6 | 54.7 | 6 | 6 | 28.3 | 4.9 |
|  | Rat | M | 280-300g | Pre | 5 | 5 | 3 | 1 | 15 | Clamp | 5 | 30 | LAD | 120 | NR | N | Pentobarbitone | N | SD |  |  |  | 6 | 51.1 | 7.4 |
| 18 | Rat | M | Adult | Pre | 15 | 10 | 1 | 2 | 30 | Clamp | 10 | 30 | LAD | 120 | NR | N | Pentobarbitone | N | SEM | 7 | 54.6 | 3.1 | 8 | 36.6 | 3 |
| 19 | Rat | M | 250-300g | Pre | 15 | 10 | 1 | 2 | 30 | Clamp | 10 | 30 | LAD | 120 | NR | N | Pentobarbitone | N | SEM | 7 | 42 | 3 | 7 | 28 | 4 |
| 20 | Rat | F | 200-250g | Per | 5 | 5 | 4 | 2 | 40 | Clamp | N/A | 45 | LAD | 120 | NR | N | Ketamine, xylazine | N | SEM | 22 | 48.7 | 3.4 | 22 | 42.2 | 3.9 |
| 21 | Pig | N/R | 20kg | Per | 5 | 5 | 4 | 1 | 20 | Tourniquet | N/A | 40 | LAD | 120 | NR | Y | Midazolam, pentobarbitone | Heparin | SEM | 10 | 60 | 5 | 10 | 38 | 5 |
| 22 | Pig | M/F | Newborn | Pre | 5 | 5 | 4 | 1 | 20 | Tourniquet | 5 | 40 | LAD | 120 | NR | N | Midazolam, azaperone, etomidate | Heparin | SD | 8 | 16.5 | 3.6 | 8 | 19.4 | 1.7 |
| 23 | Rat | M | Adult | Pre | 15 | 10 | 1 | 2 | 30 | Clamp | 10 | 30 | LAD | 120 | NR | N | Urethane | N | SEM | 6 | 55.5 | 3.1 | 6 | 21.5 | 3.5 |
| 24 | Rat | M | 8-10 weeks | Per | 10 | 10 | 1 | 1 | 10 | Tourniquet | N/A | 40 | LAD | 120 | NR | N | Pentobarbitone | N | SEM | 6 | 54.9 | 6.5 | 6 | 24.7 | 6.0 |
| 25 | Mouse | M | 12±3 weeks | Pre | 5 | 5 | 4 | 1 | 20 | Cuff | 5 | 30 | LAD | 1440 | R | Y | Ketamine, xylazine | N | SD | 5 | 37 | 4 | 5 | 17 | 3 |
| 26 | Rat | M | 250-280g | Per | 5 | 5 | 4 | 1 | 20 | Tourniquet | N/A | 45 | LAD | 4320 | R | N | Pentobarbitone | Heparin | SD | 8 | 50.5 | 4.1 | 8 | 35.6 | 4.2 |
| 27 | Rat | M | 250-350g | Pre | 15 | 0 | 1 | 2 | 30 | Clamp | 0 | 30 | LAD | 120 | NR | Y | Pentobarbitone | N | SEM | 6 | 62 | 5 | 6 | 52 | 4 |
|  | Rat | M | 250-350g | Pre | 5 | 10 | 1 | 2 | 10 | Clamp | 10 | 30 | LAD | 120 | NR | Y | Pentobarbitone | N | SEM |  |  |  | 6 | 42 | 2 |
|  | Rat | M | 250-350g | Pre | 10 | 10 | 1 | 2 | 20 | Clamp | 10 | 30 | LAD | 120 | NR | Y | Pentobarbitone | N | SEM |  |  |  | 6 | 37 | 8 |
|  | Rat | M | 250-350g | Pre | 15 | 10 | 1 | 2 | 30 | Clamp | 10 | 30 | LAD | 120 | NR | Y | Pentobarbitone | N | SEM |  |  |  | 8 | 18 | 3 |
| 28 | Rat | M | 300±25g | Pre | 5 | 5 | 3 | 1 | 15 | Clamp | 5 | 30 | LAD | 120 | NR | N | Pentobabitone | N | SD | 6 | 52.8 | 5.9 | 6 | 29.6 | 5.8 |
| 29 | Rat | M | 8 week | Per | 5 | 5 | 4 | 1 | 20 | Cuff | N/A | 45 | LAD | 120 | NR | N | Pentobabitone | Heparin | SD | 8 | 48.9 | 6.66 | 8 | 33.5 | 5.8 |
| 30 | Rat | M | 8-9 weeks | Per | 10 | N/A | 1 | 2 | 20 | Tourniquet | N/A | 30 | LAD | 180 | NR | Y | Pentobarbitone | N | SD | 9 | 60 | 3 | 9 | 48 | 1 |
| 31 | Rat | M | 250-300g | Per | 5 | 5 | 3 | 1 | 15 | Clamp | N/A | 45 | LAD | 180 | NR | N | Pentobarbitone | Heparin | SEM | 12 | 55.8 | 2.2 | 12 | 31.3 | 1.9 |
| 32 | Rat | M | 8 weeks | Per | 10 | N/A | 1 | 2 | 20 | Tourniquet | N/A | 30 | LAD | 120 | NR | Y | Pentobarbitone | N | SD | 20 | 71.6 | 8.7 | 20 | 56.9 | 8.8 |
| 33 | Rat | M | 230-260g | Pre | 5 | 5 | 3 | 1 | 15 | Clamp | 5 | 30 | LAD | 120 | NR | Y | Chloral hydrate | N | SD | 6 | 51 | 6 | 6 | 20.3 | 2.4 |
| 34 | Rat | M | Adult | Pre | 5 | 5 | 3 | 2 | 30 | Tourniquet | 5 | 30 | LAD | 180 | NR | N | Chloral hydrate | Heparin | SEM | 12 | 34.7 | 5.9 | 12 | 14.5 | 3.5 |
|  | Rat | M | Adult | Per | 5 | 5 | 3 | 2 | 30 | Tourniquet | N/A | 30 | LAD | 180 | NR | N | Chloral hydrate | Heparin | SEM |  |  |  | 12 | 15.3 | 5.2 |
|  | Rat | M | Adult | Post | 5 | 5 | 3 | 2 | 30 | Tourniquet | N/A | 30 | LAD | 180 | NR | N | Chloral hydrate | Heparin | SEM |  |  |  | 12 | 19.8 | 5.9 |

Table S4. Main characteristics of included studies.

The main characteristics included: (1) Study reference; (2) Species; (3) Gender; (4) Age or weight; (5) Conditioning protocol (pre-, per- or post-conditioning); (6) RIC cycle duration (min); (7) RIC reperfusion duration (min); (8) Number of cycles; (9) Number of limbs; (10) Total RIC ischaemia duration (min); (11) RIC occlusion technique; (12) Time between RIC and index ischaemia (min, preconditioning only); (13) Index ischaemia duration (min); (14) Coronary artery occluded; (15) Reperfusion duration (min); (16) Recovery or non-recovery; (17) Supplementary oxygen; (18) Induction anaesthetic; (19) Anticoagulants; (20) Measure of variance; (21) Control group sample size; (22) Control group mean infarct size (IS/AAR%); (23) Control group variance (IS/AAR%); (24) Conditioning group sample size; (25) Conditioning group mean infarct size (IS/AAR%); and (26) Conditioning group variance (IS/AAR%). N/R, not recorded; N/A, not applicable; LAD, left anterior descending; R, recovery; NR, non-recovery; SEM, standard error of the mean; SD, standard deviation.

**Supplementary tables**

| **Experimental factor** | **WMD** | **(95% CI)** | **% Weight** | **P-value** | **Adj R-squared** |
| --- | --- | --- | --- | --- | --- |
| Duration of isch index |  |  |  | 0.180 | 6.41% |
| 30 | 23.42 | (18.26, 28.58) | 75.16 |  |  |
| 35 | 22.0 | (9.28, 34.72) | 3.63 |  |  |
| 40 | 16.63 | (13.53, 19.73) | 21.20 |  |  |
| 45 | - | - | - |  |  |

Table S5. Meta-analysis comparing the effect of index ischaemia duration on WMD in studies investigating remote PREconditioning. N=20 comparisons; the 35 min group includes only 1 comparison.

| **Experimental factor** | **WMD** | **(95% CI)** | **% Weight** | **P-value** | **Adj R-squared** |
| --- | --- | --- | --- | --- | --- |
| Duration of isch index |  |  |  | 0.997 | -3.09% |
| 30 | 19.63 | (16.54, 22.60) | 56.44 |  |  |
| 35 | - | - | - |  |  |
| 40 | 31.24 | (29.35, 33.13) | 18.84 |  |  |
| 45 | 15.32 | (2.89, 27.75) | 24.73 |  |  |

Table S6. Meta-analysis comparing the effect of index ischaemia on WMD in studies investigating remote PERconditioning and POSTconditioning. N=16 comparisons.

| **Author (year)** | **1** | **2** | **3** | **4** | **5** | **6** | **7** | **8** | **9** | **10** | **11** | **12** | **13** | **14** | **15** | **16** | **17** | **18** | **19** | **20** | **Quality score** |
| --- | --- | --- | --- | --- | --- | --- | --- | --- | --- | --- | --- | --- | --- | --- | --- | --- | --- | --- | --- | --- | --- |
| Hibert *et al.* (2013) | 1 | 1 | 1 | 1 | 1 | 1 | 0 | 1 | 0 | 0 | 0 | 1 | 1 | 0 | 1 | 1 | 0 | 1 | 1 | 1 | 14 |
| Shahid *et al.* (2008) | 1 | 1 | 1 | 1 | 1 | 1 | 0 | 1 | 1 | 0 | 0 | 0 | 0 | 1 | 1 | 1 | 1 | 1 | 0 | 1 | 14 |
| Heinen *et al.* (2011) | 1 | 1 | 1 | 1 | 1 | 1 | 0 | 1 | 0 | 0 | 0 | 0 | 1 | 1 | 0 | 1 | 0 | 1 | 1 | 0 | 12 |
| Mastitskaya *et al.* (2012) | 1 | 1 | 1 | 1 | 1 | 0 | 1 | 1 | 0 | 0 | 0 | 0 | 0 | 0 | 1 | 1 | 0 | 0 | 1 | 1 | 11 |
| Wong *et al.* (2012) | 1 | 0 | 1 | 1 | 1 | 0 | 0 | 1 | 1 | 1 | 0 | 1 | 0 | 1 | 1 | 1 | 1 | 1 | 0 | 1 | 14 |
| Rassaf *et al.* (2014) | 1 | 1 | 1 | 1 | 0 | 0 | 0 | 0 | 1 | 0 | 0 | 0 | 0 | 1 | 1 | 1 | 0 | 1 | 1 | 1 | 11 |
| Alburquerque-Bejar *et al.* (2015) | 1 | 1 | 1 | 1 | 1 | 0 | 0 | 1 | 0 | 0 | 0 | 1 | 0 | 1 | 1 | 1 | 0 | 1 | 1 | 1 | 13 |
| Xin *et al.* (2010) | 1 | 1 | 1 | 1 | 1 | 1 | 1 | 1 | 0 | 0 | 0 | 0 | 0 | 1 | 1 | 1 | 0 | 1 | 1 | 1 | 14 |
| Sachdeva *et al.* (2014) | 1 | 1 | 1 | 1 | 1 | 1 | 0 | 1 | 0 | 1 | 0 | 1 | 0 | 1 | 1 | 1 | 1 | 1 | 0 | 1 | 15 |
| Grall *et al.* (2013) | 1 | 1 | 1 | 1 | 1 | 1 | 1 | 1 | 0 | 0 | 0 | 0 | 0 | 0 | 1 | 1 | 0 | 1 | 1 | 1 | 13 |
| Schmidt *et al.* (2006) | 1 | 1 | 1 | 1 | 1 | 1 | 1 | 1 | 0 | 1 | 1 | 0 | 1 | 1 | 1 | 1 | 1 | 1 | 1 | 1 | 18 |
| Hausenloy *et al.* (2012) | 1 | 1 | 1 | 1 | 1 | 1 | 0 | 1 | 0 | 0 | 0 | 0 | 0 | 1 | 1 | 1 | 1 | 1 | 1 | 1 | 14 |
| Zhang *et al.* (2013) | 1 | 1 | 1 | 1 | 1 | 1 | 1 | 1 | 1 | 1 | 1 | 0 | 1 | 1 | 1 | 1 | 0 | 1 | 1 | 1 | 18 |
| Li *et al.* (2009) | 1 | 1 | 1 | 1 | 1 | 1 | 0 | 1 | 0 | 0 | 0 | 0 | 0 | 1 | 1 | 1 | 0 | 1 | 1 | 1 | 13 |
| Jeanneteau *et al.* (2012) | 0 | 1 | 1 | 1 | 1 | 1 | 0 | 1 | 0 | 0 | 0 | 0 | 0 | 0 | 1 | 1 | 0 | 1 | 1 | 1 | 11 |
| Lu *et al.* (2012) | 1 | 1 | 1 | 1 | 1 | 1 | 0 | 1 | 0 | 0 | 0 | 0 | 0 | 1 | 1 | 1 | 1 | 1 | 1 | 1 | 14 |
| Weinbrenner *et al.* (2002) | 1 | 1 | 1 | 1 | 1 | 0 | 0 | 1 | 0 | 0 | 0 | 0 | 0 | 1 | 1 | 1 | 1 | 0 | 1 | 1 | 12 |
| Andreka *et al.* (2007) | 1 | 1 | 1 | 1 | 1 | 1 | 0 | 1 | 0 | 0 | 0 | 1 | 0 | 0 | 1 | 1 | 0 | 1 | 1 | 1 | 13 |
| Basalay *et al.* (2012) | 1 | 1 | 1 | 1 | 1 | 0 | 0 | 1 | 0 | 0 | 0 | 0 | 0 | 0 | 1 | 1 | 0 | 0 | 1 | 1 | 10 |
| Yu *et al.* (2014) | 1 | 1 | 1 | 1 | 1 | 1 | 1 | 1 | 1 | 0 | 0 | 0 | 0 | 1 | 1 | 1 | 0 | 0 | 1 | 1 | 14 |
| Zhu *et al.* (2013) | 1 | 1 | 1 | 1 | 1 | 1 | 1 | 1 | 0 | 0 | 0 | 0 | 0 | 1 | 1 | 1 | 1 | 1 | 1 | 1 | 15 |
| Wei *et al.* (2011) | 1 | 1 | 1 | 1 | 1 | 1 | 1 | 1 | 1 | 0 | 0 | 0 | 0 | 0 | 1 | 1 | 0 | 1 | 1 | 1 | 14 |
| Tamareille *et al.* (2011) | 1 | 1 | 1 | 1 | 1 | 1 | 1 | 1 | 0 | 0 | 1 | 0 | 0 | 0 | 1 | 1 | 0 | 1 | 1 | 1 | 14 |
| Kalakech *et al.* (2014) | 1 | 1 | 1 | 1 | 1 | 1 | 0 | 1 | 0 | 0 | 0 | 0 | 1 | 0 | 1 | 1 | 0 | 1 | 1 | 1 | 13 |
| Kalakech *et al.* (2013) | 1 | 1 | 1 | 1 | 1 | 1 | 1 | 1 | 0 | 0 | 0 | 0 | 1 | 0 | 1 | 1 | 1 | 1 | 1 | 1 | 15 |
| Lim *et al.* (2010) | 1 | 1 | 1 | 1 | 1 | 1 | 0 | 1 | 0 | 0 | 1 | 0 | 0 | 1 | 1 | 1 | 0 | 1 | 1 | 1 | 14 |
| Kiss *et al.* (2014) | 1 | 1 | 1 | 1 | 1 | 1 | 0 | 1 | 0 | 0 | 1 | 0 | 0 | 1 | 1 | 1 | 0 | 1 | 1 | 1 | 14 |
| Kharbanda *et al.* (2002) | 1 | 1 | 1 | 1 | 1 | 1 | 0 | 1 | 0 | 0 | 1 | 1 | 1 | 1 | 1 | 1 | 0 | 0 | 1 | 1 | 15 |
| Xu *et al.* (2015) | 1 | 1 | 1 | 1 | 1 | 1 | 1 | 1 | 0 | 1 | 1 | 0 | 1 | 1 | 1 | 1 | 0 | 1 | 1 | 1 | 17 |
| Zhang *et al.* (2006) | 1 | 1 | 1 | 1 | 1 | 0 | 0 | 1 | 0 | 0 | 0 | 0 | 0 | 1 | 1 | 1 | 0 | 0 | 0 | 1 | 10 |
| Cellier *et al.* (2015) | 0 | 1 | 1 | 0 | 1 | 0 | 0 | 1 | 0 | 0 | 0 | 1 | 0 | 0 | 1 | 1 | 0 | 1 | 0 | 0 | 8 |

Table S7. Study quality based on ARRIVE guidelines. Study quality items are: (1) Title; (2) Abstract; (3) Background; (4) Objectives; (5) Ethical statement; (6) Study design; (7) Experimental procedures; (8) Experimental animals; (9) Housing and husbandry; (10) Sample size; (11) Allocating animals to experimental group; (12) Experimental outcomes; (13) Statistical methods; (14) Baseline data; (15) Numbers analysed; (16) Outcomes and estimation; (17) Adverse events; (18) Interpretation/scientific implications; (19) Generalizability/translation; and (20) Funding.

| **Author (year)** | **1** | **2** | **3** | **4** | **5** | **6** | **7** | **8** | **9** | **10** | **11** | **12** | **Quality score** |
| --- | --- | --- | --- | --- | --- | --- | --- | --- | --- | --- | --- | --- | --- |
| Hibert *et al.* (2013) | 1 | 1 | 0 | 1 | 1 | 1 | 0 | 0 | 0 | 0 | 0 | 1 | 6 |
| Shahid *et al.* (2008) | 1 | 1 | 1 | 1 | 1 | 1 | 1 | 1 | 1 | 0 | 0 | 0 | 9 |
| Heinen *et al.* (2011) | 1 | 1 | 0 | 1 | 1 | 1 | 1 | 0 | 1 | 0 | 0 | 0 | 7 |
| Mastitskaya *et al.* (2012) | 1 | 0 | 0 | 1 | 1 | 1 | 1 | 1 | 1 | 0 | 0 | 1 | 8 |
| Wong *et al.* (2012) | 1 | 0 | 0 | 1 | 1 | 1 | 1 | 0 | 1 | 1 | 0 | 1 | 8 |
| Rassaf *et al.* (2014) | 1 | 0 | 0 | 1 | 0 | 0 | 0 | 0 | 0 | 0 | 0 | 1 | 3 |
| Alburquerque-Bejar *et al.* (2015) | 1 | 0 | 0 | 1 | 0 | 1 | 1 | 1 | 0 | 0 | 0 | 1 | 6 |
| Xin *et al.* (2010) | 1 | 0 | 1 | 1 | 1 | 1 | 1 | 0 | 1 | 0 | 0 | 1 | 8 |
| Sachdeva *et al.* (2014) | 1 | 1 | 0 | 1 | 0 | 1 | 1 | 0 | 0 | 0 | 0 | 1 | 6 |
| Grall *et al.* (2013) | 1 | 1 | 0 | 1 | 1 | 1 | 0 | 0 | 0 | 0 | 0 | 1 | 6 |
| Schmidt *et al.* (2006) | 1 | 1 | 1 | 1 | 1 | 1 | 1 | 1 | 1 | 1 | 1 | 0 | 11 |
| Hausenloy *et al.* (2012) | 1 | 1 | 0 | 1 | 0 | 0 | 1 | 1 | 1 | 0 | 0 | 1 | 7 |
| Zhang *et al.* (2013) | 1 | 1 | 1 | 1 | 0 | 1 | 1 | 1 | 1 | 1 | 0 | 0 | 9 |
| Li *et al.* (2009) | 1 | 1 | 0 | 1 | 1 | 1 | 1 | 0 | 1 | 0 | 0 | 0 | 7 |
| Jeanneteau *et al.* (2012) | 1 | 1 | 0 | 1 | 1 | 1 | 0 | 0 | 0 | 0 | 0 | 1 | 6 |
| Lu *et al.* (2012) | 1 | 1 | 0 | 1 | 1 | 1 | 1 | 0 | 1 | 0 | 0 | 0 | 7 |
| Weinbrenner *et al.* (2002) | 1 | 0 | 0 | 1 | 1 | 1 | 1 | 1 | 1 | 0 | 0 | 0 | 7 |
| Andreka *et al.* (2007) | 1 | 1 | 1 | 1 | 1 | 1 | 1 | 0 | 0 | 0 | 0 | 1 | 8 |
| Basalay *et al.* (2012) | 1 | 0 | 0 | 1 | 1 | 1 | 1 | 1 | 1 | 0 | 0 | 0 | 7 |
| Yu *et al.* (2014) | 1 | 1 | 0 | 1 | 0 | 0 | 1 | 0 | 1 | 0 | 0 | 0 | 5 |
| Zhu *et al.* (2013) | 1 | 1 | 1 | 1 | 1 | 0 | 1 | 0 | 0 | 0 | 0 | 1 | 7 |
| Wei *et al.* (2011) | 1 | 1 | 1 | 1 | 1 | 0 | 1 | 0 | 0 | 0 | 0 | 1 | 7 |
| Tamareille *et al.* (2011) | 1 | 1 | 1 | 1 | 1 | 1 | 0 | 0 | 0 | 0 | 0 | 1 | 7 |
| Kalakech *et al.* (2014) | 1 | 1 | 1 | 1 | 1 | 1 | 0 | 0 | 0 | 0 | 0 | 1 | 7 |
| Kalakech *et al.* (2013) | 1 | 1 | 1 | 1 | 1 | 1 | 0 | 0 | 0 | 0 | 0 | 1 | 7 |
| Lim *et al.* (2010) | 1 | 1 | 0 | 1 | 1 | 1 | 1 | 0 | 1 | 0 | 0 | 0 | 7 |
| Kiss *et al.* (2014) | 1 | 1 | 0 | 1 | 1 | 1 | 1 | 0 | 1 | 0 | 0 | 1 | 8 |
| Kharbanda *et al.* (2002) | 1 | 1 | 1 | 1 | 1 | 1 | 1 | 1 | 1 | 0 | 1 | 0 | 10 |
| Xu *et al.* (2015) | 1 | 1 | 1 | 1 | 1 | 1 | 1 | 1 | 1 | 1 | 1 | 1 | 12 |
| Zhang *et al.* (2006) | 1 | 0 | 0 | 1 | 1 | 1 | 1 | 0 | 1 | 0 | 0 | 0 | 6 |
| Cellier *et al.* (2015) | 1 | 0 | 0 | 1 | 1 | 1 | 0 | 0 | 0 | 0 | 0 | 0 | 4 |

Table S8. Study quality report. Study quality items are: (1) Publication in a peer-reviewed journal; (2) Randomization to either control or conditioning treatment with RIC or placebo control; (3) Blinded assessment of outcome; (4) Statement of compliance with regulatory requirement; (5) Method of confirmation of ischemia; (6) Statement of control of temperature; (7) Statement of recording ECG; (8) Statement of measuring PaO2 or SaO2; (9) Statement of measurement of BP; (10) Sample size calculation; (11) Blinded application of conditioning protocol; and (12) Statement of conflict of interest.

References of included studies^1–31^

1. Alburquerque-Bé JJ, Barba I, Inserte J, et al. Combination therapy with remote ischaemic conditioning and insulin or exenatide enhances infarct size limitation in pigs. doi:10.1093/cvr/cvv171.

2. Andreka G, Vertesaljai M, Szantho G, et al. Remote ischaemic postconditioning protects the heart during acute myocardial infarction in pigs. *Heart*. 2007;93(6):749-752. doi:10.1136/hrt.2006.114504.

3. Basalay M, Barsukevich V, Mastitskaya S, et al. Remote ischaemic pre- and delayed postconditioning - similar degree of cardioprotection but distinct mechanisms. *Exp Physiol*. 2012;97(8):908-917. doi:10.1113/expphysiol.2012.064923.

4. Cellier L, Tamareille S, Kalakech H, et al. Remote Ischemic Conditioning Influences Mitochondrial Dynamics. *SHOCK*. 2015:1. doi:10.1097/SHK.0000000000000500.

5. Grall S, Prunier-Mirebeau D, Tamareille S, et al. ENDOPLASMIC RETICULUM STRESS PATHWAY INVOLVEMENT IN LOCAL AND REMOTE MYOCARDIAL ISCHEMIC CONDITIONING. doi:10.1097/SHK.0b013e31828e4f80.

6. Hausenloy DJ, Iliodromitis EK, Andreadou I, et al. Investigating the Signal Transduction Pathways Underlying Remote Ischemic Conditioning in the Porcine Heart. doi:10.1007/s10557-011-6364-y.

7. Heinen NM, Pütz VE, Görgens JI, et al. Cardioprotection by remote ischemic preconditioning exhibits a signaling pattern different from local ischemic preconditioning. *Shock*. 2011. doi:10.1097/SHK.0b013e31821d8e77.

8. Hibert P, Prunier-Mirebeau D, Beseme O, et al. Apolipoprotein a-I is a potential mediator of remote ischemic preconditioning. *PLoS One*. 2013;8(10):e77211. doi:10.1371/journal.pone.0077211.

9. Jeanneteau J, Hibert P, Martinez MC, et al. Microparticle release in remote ischemic conditioning mechanism. *AJP Hear Circ Physiol*. 2012;303(7):H871-H877. doi:10.1152/ajpheart.00102.2012.

10. Kalakech H, Hibert P, Prunier-Mirebeau D, et al. RISK and SAFE Signaling Pathway Involvement in Apolipoprotein A-I-Induced Cardioprotection. *PLoS One*. 2014;9(9). doi:10.1371/journal.pone.0107950.

11. Kalakech H, Tamareille S, Pons S, et al. Role of hypoxia inducible factor-1α in remote limb ischemic preconditioning. *J Mol Cell Cardiol*. 2013;65:98-104. doi:10.1016/j.yjmcc.2013.10.001.

12. Kharbanda R, Mortensen U, White P, et al. Transient Limb Ischemia Induces Remote Ischemic Preconditioning In Vivo. doi:10.1161/01.CIR.0000043806.51912.9B.

13. Kiss A, Tratsiakovich Y, Gonon AT, et al. The Role of Arginase and Rho Kinase in Cardioprotection from Remote Ischemic Perconditioning in Non-Diabetic and Diabetic Rat In Vivo. doi:10.1371/journal.pone.0104731.

14. Li C-M, Zhang X-H, Ma X-J, Luo M. Limb ischemic postconditioning protects myocardium from ischemia-reperfusion injury. *Scand Cardiovasc J*. 2006;40(5):312-317. doi:10.1080/14017430600925292.

15. Lim SY, Yellon DM, Hausenloy DJ. The neural and humoral pathways in remote limb ischemic preconditioning. doi:10.1007/s00395-010-0099-y.

16. Lu Y, Dong C-S, Yu J, Li H. Morphine reduces the threshold of remote ischemic preconditioning against myocardial ischemia and reperfusion injury in rats: the role of opioid receptors. *J Cardiothorac Vasc Anesth*. 2012;26(3):403-406. doi:10.1053/j.jvca.2011.07.036.

17. Mastitskaya S, Marina N, Gourine A, et al. Cardioprotection evoked by remote ischaemic preconditioning is critically dependent on the activity of vagal pre-ganglionic neurones. *Cardiovasc Res*. 2012;95(4):487-494. doi:10.1093/cvr/cvs212.

18. Sachdeva J, Dai W, Gerczuk PZ, Kloner RA. Combined Remote Perconditioning and Postconditioning Failed to Attenuate Infarct Size and Contractile Dysfunction in a Rat Model of Coronary Artery Occlusion. doi:10.1177/1074248413518967.

19. Schmidt MR, Smerup M, Konstantinov IE, et al. Intermittent peripheral tissue ischemia during coronary ischemia reduces myocardial infarction through a KATP-dependent mechanism: first demonstration of remote ischemic perconditioning. *Am J Physiol Heart Circ Physiol*. 2007;292(4):H1883-H1890. doi:10.1152/ajpheart.00617.2006.

20. Shahid M, Tauseef M, Sharma KK, Fahim M. Brief femoral artery ischaemia provides protection against myocardial ischaemia-reperfusion injury in rats: the possible mechanisms. *Exp Physiol*. 2008. doi:10.1113/expphysiol.2007.041442.

21. Tamareille S, Mateus V, Nehmat @bullet, et al. RISK and SAFE signaling pathway interactions in remote limb ischemic perconditioning in combination with local ischemic postconditioning. doi:10.1007/s00395-011-0210-z.

22. Tienush Rassaf,* Matthias Totzeck,* Ulrike B. Hendgen-Cotta, Sruti Shiva, Gerd Heusch MK. Circulating Nitrite Contributes to Cardioprotection by Remote Ischemic Preconditioning. *Circulation*. 2014;114:1601-1610.

23. Wei M, Xin P, Li S, et al. Repeated Remote Ischemic Postconditioning Protects Against Adverse Left Ventricular Remodeling and Improves Survival in a Rat Model of Myocardial Infarction Short Communication. doi:10.1161/CIRCRESAHA.110.236190.

24. Weinbrenner H, Nelles M, Herzog N, Sarvary L, Strasser RH. R emote preconditioning by infrarenal occlusion of the aorta protects the heart from infarction: a newly identified non-neuronal but PKC-dependent pathway. *Cardiovasc Res*. 2002;55:590-601. www.elsevier.com.

25. Wong GTC, Lu Y, Mei B, Xia Z, Irwin MG. Cardioprotection from remote preconditioning involves spinal opioid receptor activation. *Life Sci*. 2012;91(17-18):860-865. doi:10.1016/j.lfs.2012.08.037.

26. Xin P, Zhu W, Li J, et al. Combined local ischemic postconditioning and remote perconditioning recapitulate cardioprotective effects of local ischemic preconditioning.

27. Xu Y, Li R, Xue F, et al. κ‑Opioid receptors are involved in enhanced cardioprotection by combined fentanyl and limb remote ischemic postconditioning. *J Anesth*. 1998;29:535-543. doi:10.1007/s00540-015-1998-8.

28. Yu Y, Jia X, Zong Q, et al. Remote ischemic postconditioning protects the heart by upregulating ALDH2 expression levels through the PI3K/Akt signaling pathway. *Mol Med Rep*. 2014;10(1):536-542. http://www.spandidos-publications.com/mmr/10/1/536/abstract. Accessed September 15, 2015.

29. Zhang J, Wang Q, Xue F, et al. Ischemic preconditioning produces more powerful anti-inflammatory and cardioprotective effects than limb remote ischemic postconditioning in rats with myocardial ischemia-reperfusion injury. *Chin Med J (Engl)*. 2013;126(20):3949-3955. http://www.ncbi.nlm.nih.gov/pubmed/24157164. Accessed September 29, 2015.

30. Zhang S-Z, Wang N-F, Xu J, et al. ␬-Opioid Receptors Mediate Cardioprotection by Remote Preconditioning. *Anesthesiology*. 2006;105:550-556.

31. Zhu S-B, Yong Liu I, Yu Zhu I, et al. Remote preconditioning, perconditioning, and post- conditioning: a comparative study of their cardio- protective properties in rat models. doi:10.6061/clinics/2013(02)OA22.

Reference List

1. Flores-Mir C, Major MP, Major PW. Search and selection methodology of systematic reviews in orthodontics (2000-2004). American journal of orthodontics and dentofacial orthopedics : official publication of the American Association of Orthodontists, its constituent societies, and the American Board of Orthodontics. 2006; **130**(2): 214-7.

2. Major MP, Major PW, Flores-Mir C. An evaluation of search and selection methods used in dental systematic reviews published in English. J Am Dent Assoc. 2006; **137**(9): 1252-7.

3. Major MP, Major PW, Flores-Mir C. Benchmarking of reported search and selection methods of systematic reviews by dental speciality. Evidence-based dentistry. 2007; **8**(3): 66-70.

4. Consumers and Communication Group resources for authors. 2013 [cited 2015 12 August]; Available from: <http://cccrg.cochrane.org/author-resources>

5. Liberati A, Altman DG, Tetzlaff J, Mulrow C, Gotzsche PC, Ioannidis JP, et al. The PRISMA statement for reporting systematic reviews and meta-analyses of studies that evaluate healthcare interventions: explanation and elaboration. BMJ. 2009; **339**: b2700.

6. O’Connor D, Green S, Higgins JPT. Chapter 5: Defining the review question and developing criteria for including studies. In: Higgins JPT, Green S, editors. Cochrane handbook for systematic reviews of interventions version 510: The Cochrane Collaboration; 2011.

7. Hood WB, Jr., McCarthy B, Lown B. Myocardial infarction following coronary ligation in dogs. Hemodynamic effects of isoproterenol and acetylstrophanthidin. Circ Res. 1967; **21**(2): 191-9.

8. Ytrehus K, Liu Y, Tsuchida A, Miura T, Liu GS, Yang XM, et al. Rat and rabbit heart infarction: effects of anesthesia, perfusate, risk zone, and method of infarct sizing. Am J Physiol. 1994; **267**(6 Pt 2): H2383-90.

9. Liu YH, Yang XP, Nass O, Sabbah HN, Peterson E, Carretero OA. Chronic heart failure induced by coronary artery ligation in Lewis inbred rats. Am J Physiol. 1997; **272**(2 Pt 2): H722-7.

10. Hodgin JB, Maeda N. Minireview: estrogen and mouse models of atherosclerosis. Endocrinology. 2002; **143**(12): 4495-501.

11. Murphy E, Steenbergen C. Gender-based differences in mechanisms of protection in myocardial ischemia-reperfusion injury. Cardiovasc Res. 2007; **75**(3): 478-86.

12. Black SC, Rodger IW. Methods for studying experimental myocardial ischemic and reperfusion injury. Journal of pharmacological and toxicological methods. 1996; **35**(4): 179-90.

13. Redfors B, Shao Y, Omerovic E. Influence of anesthetic agent, depth of anesthesia and body temperature on cardiovascular functional parameters in the rat. Laboratory animals. 2014; **48**(1): 6-14.

14. Kato R, Foex P. Myocardial protection by anesthetic agents against ischemia-reperfusion injury: an update for anesthesiologists. Canadian journal of anaesthesia = Journal canadien d'anesthesie. 2002; **49**(8): 777-91.

15. Kottenberg E, Thielmann M, Bergmann L, Heine T, Jakob H, Heusch G, et al. Protection by remote ischemic preconditioning during coronary artery bypass graft surgery with isoflurane but not propofol - a clinical trial. Acta anaesthesiologica Scandinavica. 2012; **56**(1): 30-8.

16. Kottenberg E, Musiolik J, Thielmann M, Jakob H, Peters J, Heusch G. Interference of propofol with signal transducer and activator of transcription 5 activation and cardioprotection by remote ischemic preconditioning during coronary artery bypass grafting. The Journal of thoracic and cardiovascular surgery. 2014; **147**(1): 376-82.

17. Reimer KA, Lowe JE, Rasmussen MM, Jennings RB. The wavefront phenomenon of ischemic cell death. 1. Myocardial infarct size vs duration of coronary occlusion in dogs. Circulation. 1977; **56**(5): 786-94.

18. Tsutsumi YM, Patel HH, Lai NC, Takahashi T, Head BP, Roth DM. Isoflurane produces sustained cardiac protection after ischemia-reperfusion injury in mice. Anesthesiology. 2006; **104**(3): 495-502.

19. McGrath JC, Drummond GB, McLachlan EM, Kilkenny C, Wainwright CL. Guidelines for reporting experiments involving animals: the ARRIVE guidelines. British journal of pharmacology. 2010; **160**(7): 1573-6.

20. Kilkenny C, Browne W, Cuthill IC, Emerson M, Altman DG. Animal research: reporting in vivo experiments: the ARRIVE guidelines. British journal of pharmacology. 2010; **160**(7): 1577-9.

21. Macleod MR, O'Collins T, Howells DW, Donnan GA. Pooling of animal experimental data reveals influence of study design and publication bias. Stroke; a journal of cerebral circulation. 2004; **35**(5): 1203-8.
